# Effectiveness of Total Body Resistance Exercise Intervention on Sleep Quality Among Nursing Staff

**DOI:** 10.1097/jnr.0000000000000739

**Published:** 2026-03-25

**Authors:** Li-Chun YANG, Ke-Hsin CHUEH, Bih-O LEE, Chien-Chang HO, Peng-Cheng HSU

**Affiliations:** 1 Department of Nursing, Fu Jen Catholic University Hospital, New Taipei City, Taiwan; 2 Department of Nursing, College of Medicine, Fu Jen Catholic University, New Taipei City, Taiwan; 3 Bachelor Degree Program of Long-Term Care and Health Management, School of Continuing Education, Fu Jen Catholic University, New Taipei City, Taiwan; 4 Chung Hwa University of Medical Technology, Tainan City, Taiwan; 5 Sports Medicine Center, Fu Jen Catholic Hospital, New Taipei City, Taiwan; 6 Center for Health Research and Innovation, Fu Jen Catholic University, New Taipei City, Taiwan; 7 Department of Physical Education, and Ph.D. Program in Nutrition and Food Science, Fu Jen Catholic University, New Taipei City, Taiwan

**Keywords:** nursing staff, sleep quality, total resistance exercise (TRX), quasi-experimental study

## Abstract

**Background:**

The sleep quality of nursing staff is a critical issue worldwide due to the significant impact of their unique work patterns on public health and their job performance. However, research on the efficacy of exercise interventions in terms of improving sleep quality in nurses is inadequate.

**Purpose:**

This study was designed to explore the effects of a total resistance exercise (TRX) training program performed twice a week on improving the sleep quality of nursing staff.

**Methods:**

A quasi-experimental design was employed, and a total of 65 nursing staff were enrolled as participants. The experimental group (33 participants) participated in a 12-week TRX training program, conducted twice a week for 90 min per session. The control group (32 participants) received no intervention. Sleep quality was assessed using the Chinese version of the Pittsburgh Sleep Quality Index (PSQI) at baseline, week 6, and week 12. Generalized estimating equations were used to evaluate the effectiveness of the intervention.

**Results:**

The TRX training program demonstrated positive effects in terms of improving overall sleep quality, enhancing subjective sleep quality, and alleviating daytime dysfunction. These results demonstrate a positive impact of TRX on sleep quality in nurses throughout the intervention period.

**Conclusions:**

The findings of this study highlight the potential benefits of implementing a 12-week TRX exercise intervention for nursing staff. It is recommended that hospital management consider incorporating this or a similar exercise program into comprehensive health promotion strategies to enhance the sleep health of nursing personnel.

## Introduction

Shift work has a direct influence on the sleep quality of nursing staff and an indirect influence on their occupational burnout and job performance (Leyva-Vela et al., 2018). The unique demands of the nursing role render the scheduling of appropriate and sufficient exercises challenging, potentially leading to sleep deprivation or disturbances that may lead to clinical decision errors that jeopardize patient safety and increase institutional expenses (Scott et al., 2014).

Total resistance exercise (TRX) is a popular modern training method that employs suspension techniques and body weight as resistance. By adjusting body tilt angles, individuals can effectively control exercise intensity, thereby engaging multiple muscle groups to enhance strength, cardiovascular fitness, balance, coordination, and especially core stability (Naderi et al., 2023; Smith et al., 2016). These characteristics make TRX particularly suitable for nursing professionals, whose work involves physically demanding tasks such as patient lifting and prolonged standing. The adjustable intensity of TRX exercises—achieved through simple changes in body angle—offers a time-efficient, low-equipment training option that is adaptable to the needs of irregular schedules. This functional alignment supports the potential for using TRX to alleviate sleep disturbances and occupational stress among nurses.

The mechanisms by which TRX training may improve sleep, as hypothesized in this study, include stress reduction via endorphin release, circadian rhythm regulation through consistent exercise timing, and enhanced neuromuscular relaxation due to its core-focused, functional movements (Boecker et al., 2008). Regular physical activity is known to elevate endogenous endorphins, which help reduce stress and promote psychological well-being, thereby facilitating better sleep (Kredlow et al., 2015). Moreover, both aerobic and resistance training have been shown to play a role in circadian rhythm entrainment, contributing to more consistent and restorative sleep patterns (Youngstedt et al., 2002). TRX, as a resistance-based modality, may further support sleep onset and maintenance, particularly in populations experiencing occupational strain, such as nurses, by reducing muscular tension and sympathetic arousal (Dolezal et al., 2017). These mechanisms collectively support the theoretical rationale for the hypothesis in this study.

Growing evidence supports the role of resistance training in improving sleep and mental health outcomes in shift workers. For instance, Kovacevic et al. (2018) reported that an 8-week resistance training program significantly improved sleep quality, reduced fatigue, and alleviated psychological stress among shift-working nurses. Amiri et al. (2021) demonstrated that combining regular exercise with sleep hygiene education effectively enhanced sleep quality and daytime functioning in rotating-shift workers. Furthermore, the findings of systematic reviews confirm that resistance training improves sleep quality and reduces the symptoms of anxiety and depression (Kovacevic et al., 2018). Moderate-to-vigorous physical activity has also been associated with faster sleep onset, fewer nighttime awakenings, and better overall sleep efficiency (D’Aurea et al., 2018; Podhorecka et al., 2017).

Among moderate-intensity exercise interventions, TRX stands out for its focus on core stability, functional movement, and group dynamics. These elements support postural control and neuromuscular coordination, which are critical to the physically demanding tasks engaged in by nurses, while enhancing motivation and adherence. Compared with traditional exercises, TRX may better meet the occupational and health needs of nurses. However, evidence specifically related to TRX and sleep efficacy in nurses remains limited. This study was designed to address this gap by evaluating the long-term effects of a 12-week TRX program on sleep quality among hospital nurses with the goal of informing future workplace health promotion strategies.

### Literature Review

A substantial proportion of nursing personnel average <7 hr of sleep before work, with a significant correlation reported between reduced sleep duration and diminished nursing care quality and patient safety (Stimpfel et al., 2020). Based on prior research, insufficient sleep, a common issue for night-shift nurses, can lead to impairments in cognitive functions essential for nursing, thus significantly impacting the quality of care and patient safety (Almhdawi et al., 2021). Research examining the health of registered nurses and their irregular sleep patterns has revealed that deteriorating sleep quality and decreasing sleep durations contribute to various health risks, such as metabolic syndrome, and ultimately lead to sleep disorders characterized by fatigue and drowsiness, as well as to other serious work-related consequences such as impaired judgment (Zhang et al., 2023). Given the exceptional demands, intensity, and stress inherent to nursing work, compounded by the necessity of night shifts, rotational shifts, and continuous acquisition of advanced medical knowledge and techniques, the compromised physical and mental states of nursing personnel can precipitate occupational burnout, further exacerbating sleep quality and rendering nurses susceptible to sleep disorders (Carthon et al., 2021).

TRX, a suspension-based resistance training modality, leverages the instability of ropes to enhance muscular endurance; strengthen core muscles; improve balance, coordination, and flexibility; and stabilize joints. Because it employs body weight and modulates the vertical angle of the lever arm relative to the ground to adjust training intensity, TRX may also serve as an aerobic exercise regimen (Gulmez, 2017). TRX permits customization based on individual fitness goals and capacities, enabling the design of personalized movements that progressively increase exercise intensity, duration, and frequency. When implemented using periodic training cycles with distinct goals and methodologies, TRX can help optimize exercise performance. The TRX regimen designed by Pratt (2015) incorporates squats, push-ups, sit-ups, rowing, and planks into six unique exercises per training session with 6–20 repetitions for each exercise and twice-weekly training sessions.

The findings of prior systematic reviews demonstrate that both resistance training and core stability training contribute to improved sleep quality, particularly in terms of reducing sleep onset latency, increasing deep sleep duration, and enhancing overall sleep efficiency, indicating that exercise interventions have a positive impact on sleep (Amiri et al., 2021; Cao et al., 2020; Kovacevic et al., 2018; Yue et al., 2022). Also, a growing body of literature supports a positive relationship between exercise and sleep quality. Both subjective and objective improvements in sleep have been observed following regular physical activity, with moderate- to high-intensity exercise showing particular efficacy, especially in adults with sleep disturbances (Kline et al., 2021; Lowe et al., 2019). Among various exercise types, resistance training has been consistently identified as a promising intervention for enhancing sleep quality. A systematic review by Kovacevic et al. (2018) confirmed that resistance exercise significantly improves sleep parameters, especially subjective sleep quality, and indirectly alleviates anxiety and depression through improved sleep outcomes. In addition, Wang et al. (2021) reported moderate-intensity resistance training may even outperform high-intensity training in enhancing sleep quality while also reducing comorbid conditions such as depression and cardiovascular risks. Collectively, these findings establish resistance training as an accessible, safe, and effective nonpharmacological strategy for improving sleep health, with potential benefits extending to both physical and mental well-being.

The several advantages of TRX training relevant to nurses include: (1) limited time requirement (around 30 min); (2) minimal equipment and space requirement, making it feasible for implementation in hospital and home settings; and (3) adjustable intensity through body position adjustments, making it suited to individuals across varying levels of fitness. For example, the implementation in Katsanis et al. (2021) of a suspension-based resistance training program in a school setting highlighted the practicality of TRX in terms of its minimal equipment and space requirements and adjustable intensity through changes in body angle to accommodate the diverse physical capacities of adolescents. Similarly, Gaedtke and Morat (2015) evaluated the feasibility of a 12-week TRX suspension training intervention for healthy older adults and demonstrated the progressive adjustment of training intensity by modifying body posture at each stage, supporting the applicability of TRX across populations with varying fitness conditions. In summary, TRX suspension training appears to offer strong practical feasibility due to its time efficiency, minimal space and equipment requirements, and adjustable intensity. Furthermore, current evidence suggests its potential benefits in enhancing muscular strength, balance, and sleep quality.

Based on the findings in the literature, nurses working in general wards and intensive care units (ICUs) frequently experience poor sleep quality (Lin et al., 2015; Tsai et al., 2019). However, studies on the efficacy of exercise interventions in enhancing sleep quality in nurses are limited. The primary challenges impeding the establishment of regular exercise routines among nursing staff include difficulties in scheduling exercise time due to shift work and a perceived lack of interest in available exercise options. Therefore, in this study, the authors collaborated with sports and nursing experts to design a suitable full-body TRX regimen and then empirically evaluated its impact on sleep quality in nursing staff. Despite the growing evidence supporting the benefits of resistance training on sleep quality, to the authors’ knowledge, no studies in the literature have reported on the effects of TRX with regard to sleep quality in nurses. Therefore, this study was designed to bridge this gap by evaluating the feasibility and effectiveness of using a TRX-based intervention to improve the subjective sleep quality of nurses.

## Methods

### Study Design and Sample

**Figure 1:**
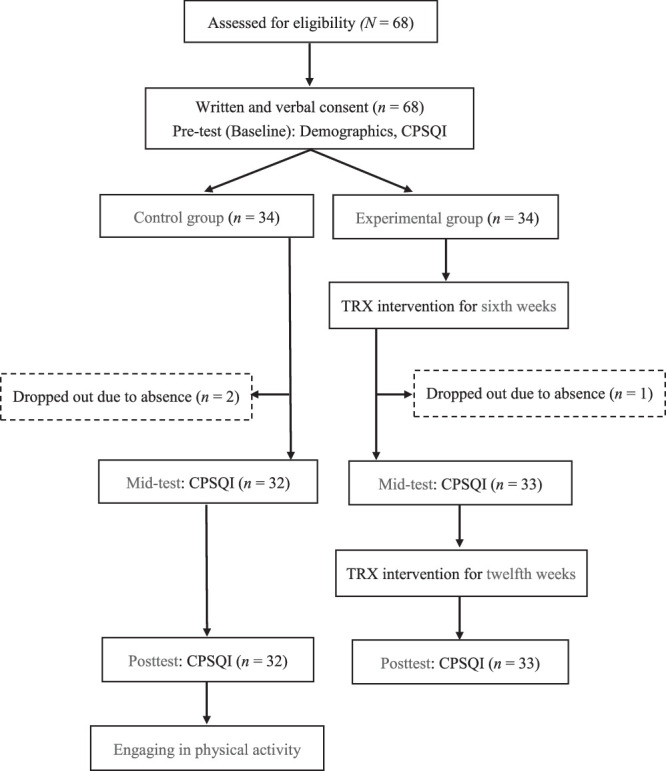
*Research Design Flowchart and Participant Allocation* Note. CPSQI = Chinese Pittsburgh Sleep Quality Index.

A quasi-experimental design with repeated measures was employed in this study. The participants were nursing staff from a regional teaching hospital in northern Taiwan. Data from nursing staff aged at least 20 years with a minimum of 3 months of hospital employment and who voluntarily agreed to participate were included in the analysis. The exclusion criteria included pregnancy and prior engagement in TRX or gym activities. Given an α value of .05, a *B* value of 0.8, an effect size of 0.25, and an estimated dropout rate of 20%, a minimum sample of 28 participants was required. Ultimately, this study included 68 participants, 34 of whom were assigned, respectively, to the experimental and control groups. The study design flowchart and participant distribution are presented in Figure 1.

The study protocol was approved by the hospital’s institutional review board (IRB: FJUH111187). All of the participants provided informed written consent. In the event of injury sustained during TRX training, the principal investigator would accompany the injured participant to the sports medicine outpatient clinic, with medical costs covered by the National Health Insurance. Any participant injured during the course of this study would be required to end their participation. To adhere to ethical research principles, upon completion of this study, the control group received a TRX training session and a 15-min office worker health exercise video provided by the Health Promotion Administration, Ministry of Health and Welfare.

Before the study commencement, the upper management of the hospital’s nursing department was invited to attend a briefing session. With approval from the nursing unit supervisor, potential participants were informed of the study objectives, procedures, and relevant details. Subsequently, 68 nursing staff were selected. To maintain anonymity, the participants were assigned numerical identifiers and randomly paired. The male and female participants were segregated and randomly allocated to either the experimental or control group at a 1:1 gender ratio. Participant data were collected using the Chinese version of the Pittsburgh Sleep Quality Index (CPSQI) at three time points: pretest (week 0), mid-test (week 6), and posttest (week 12). A triple-blind approach was implemented, ensuring that the participants, instructors, and investigator were all blinded to group allocation.

The experimental group participated in the 12-week TRX training program at a specialized university fitness center classroom from October 1, 2022, to January 13, 2023. Baseline data were collected one week before the intervention, mid-test data were obtained during the sixth week of the intervention, and posttest data were collected 1 week after completing the intervention. A certified TRX instructor tailored the training regimen to the needs of each participant and provided in-person supervision during each session. Training sessions were conducted twice weekly, with each session lasting 90 min.

The TRX training regimen incorporated a progressive overload approach to enhance performance that gradually increased resistance, exercise intensity, duration, or frequency. The training program was structured into different phases to optimize performance: weeks 1–3 focused on movement pattern establishment, weeks 4–6 emphasized muscular endurance, weeks 7–9 targeted muscle hypertrophy, and weeks 10–12 prioritized strength building. Proper movement execution is essential to preventing injuries, and compensatory movements necessitate load reduction or increased stability to maintain correct form. Each TRX session commenced with a 5-min warm-up and concluded with a 5-min cool-down. The training regimen comprised three sets of six exercises, with 5–10 s of rest between exercises and 2–3 min of rest between sets for participant recovery and hydration. Post exercise, the participants engaged in cool-down activities, including stretching and relaxation of targeted muscle groups to facilitate lactic acid clearance and injury prevention. The control group maintained their customary physical activities as per their normal routine. Both groups were asked to otherwise maintain their usual physical activity, exercise habits, workload, and lifestyle.

### Outcome Measures

The research instruments employed in this study included a demographic information survey and the CPSQI.

#### Sleep quality

The original PSQI, developed in 1989 by Buysse and colleagues, is designed to assess subjective sleep experiences over the previous 1-month period. This instrument comprises seven components: subjective sleep quality, sleep latency, sleep duration, sleep efficiency, sleep disturbances, use of sleep medication, and daytime dysfunction. Each item is rated on a 0–3 scale, yielding a total possible index score ranging from 0 to 21, with scores <6 indicating favorable sleep quality and higher scores indicating relatively poor sleep quality (Buysse et al., 1989). In this study, the traditional Chinese version of the CPSQI translated by Tsai et al. (2005) was used, which earned a Cronbach’s α of .77 in the original study and was .623 in this study.

The demographic data collected from participants included gender, age, workplace unit, years of service, height, weight, and body mass index.

### Data Analysis

Descriptive and inferential statistical analyses, conducted using IBM SPSS 25.0 (IBM Corp., Armonk, NY, USA), were used to examine the differences in demographic characteristics and overall sleep quality between the experimental and control groups. Also, generalized estimating equations (GEEs) were employed to assess the effectiveness of the intervention, with all statistical tests being two-tailed and the significance level set at *p*<.05.

## Results

Given the concurrent COVID-19 pandemic (Taiwan Centers for Disease Control, 2022), the participants were permitted to request leave and postpone TRX training if infected. Due to one experimental group participant’s severe illness following their COVID-19 infection in week 3 and two control group participants’ failing to adhere to measurement schedules in week 6 due to personal reasons, these individuals were excluded from the study. Thus, 65 participants completed the 12-week study, with the experimental and control groups comprising 33 and 32 participants, respectively, yielding an overall completion rate of 95.59%. The experimental group attended an average of 20.94 out of 24 sessions (*SD*=4.80), representing an attendance rate of 87.25%.

As shown in Table 1, the sample was predominantly female (*n*=56, 86.2%). Most of the participants were aged 20–25 years (*n*=38, 58.5%), followed by those who were aged 26–30 years (*n*=20, 30.8%). A majority were employed in general wards (*n*=36, 55.4%). The mean duration of service was 3.31 (*SD*=2.87) years. The independent sample *t* tests, chi-squared tests, and Fisher’s exact tests revealed no significant differences between the two groups in terms of gender, age, workplace unit, years of service, height, weight, or body mass index (*p*>.05), confirming homogeneity in participant demographics.

**Table 1:** Participant Demographics, Experimental, and Control Groups

Variable	Total (*N*=65)	Experimental Group (*n*=33)	Control Group (*n*=32)	*p*
Gender ^a^				.733
Male	9 (13.8)	4 (12.1)	5 (15.6)	
Female	56 (86.2)	29 (87.9)	27 (84.4)	
Age (years) ^b^				.160
20–25	38 (58.5)	16 (48.5)	22 (68.8)	
26–30	20 (30.8)	13 (39.4)	7 (21.9)	
31–35	3 (4.6)	2 (6.1)	1 (3.1)	
36–40	1 (1.5)	0 (0.0)	1 (3.1)	
41–45	2 (3.1)	2 (6.1)	0 (0.0)	
46–50	1 (1.5)	0 (0.0)	1 (3.1)	
Workplace unit ^b^				.102
General ward	36 (55.4)	15 (45.5)	21 (65.6)	
Intensive care unit	29 (44.6)	18 (54.5)	11 (34.4)	
Variable	*M*±*SD*	*M*±*SD*	*M*±*SD*	
Years of service ^c^	3.31±2.87	3.71±3.21	2.89±2.44	.251
Height (cm) ^c^	161.09±7.35	160.39±6.46	161.81±8.22	.441
Weight (kg) ^c^	60.05±13.35	62.39±13.86	58.51±12.85	.246
Body mass index ^c^	23.08±4.71	23.55±5.86	22.34±4.85	.368

*Note.* Categorical variables presented as numbers (percentages); continuous variables presented as means±standard deviations.

^a^ Fisher’s exact test. ^b^ χ^2^ test. ^c^ Independent samples *t* test.

**Figure 2:**
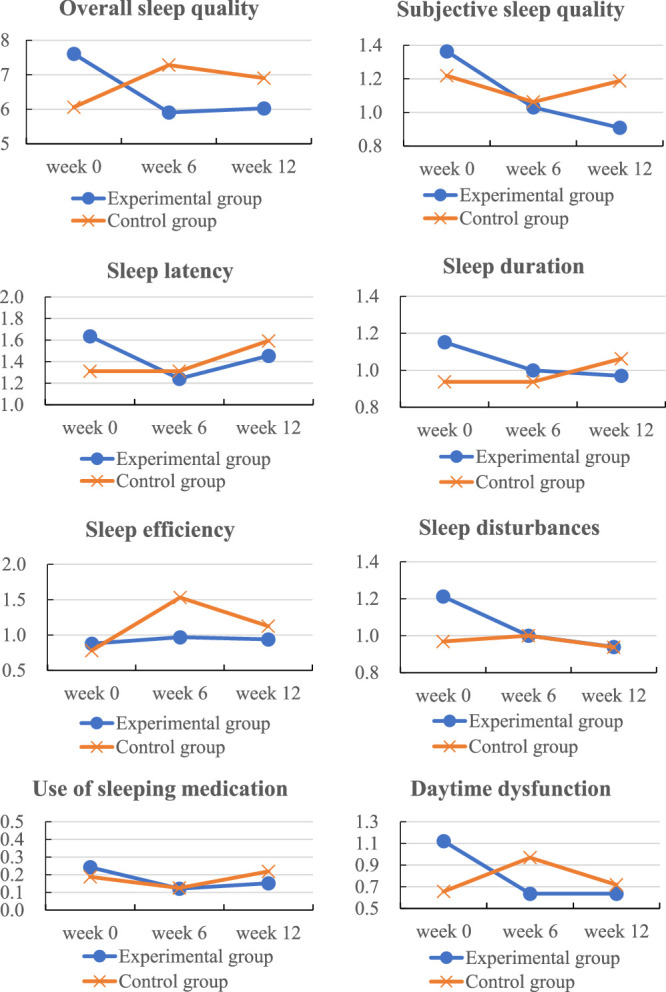
Changes in Overall Sleep Quality and Subcomponents Over Time in the Experimental and Control Groups

As shown in Table 2, at pretest, the experimental group exhibited significantly poorer overall sleep quality than the control group (7.61±3.14 vs. 6.06±2.45, *t*=2.20, *p*=.031). An overall sleep quality and seven components analysis was performed using GEEs, with the results presented in Table 3 and Figure 2. In terms of overall sleep quality, significant interactions (*p*<.05) were revealed between the experimental group and both the mid-test (*B*=−2.92, *p*<.001) and posttest (*B*=−2.42, *p*=.001) results for changes in overall sleep quality, indicating significantly different changes in overall sleep quality from the pretest to the posttest between the two groups. In the experimental group, there was a notable decrease in overall sleep quality between pretest and mid-test, followed by a slight increase from mid-test to posttest. However, in the control group, there was a notable increase in overall sleep quality between pretest and mid-test, followed by a slight decrease from mid-test to posttest. The more pronounced overall decrease in the experimental group suggests a greater improvement in overall sleep quality compared with the control group, supporting the efficacy of TRX in enhancing overall sleep quality.

**Table 2:** Descriptive Statistics for the Overall Sleep Quality and Subcomponents in the Experimental and Control Groups at Different Time Points (*N*=65)

Variable	Total	*M*±*SD*		*t*	*p*
		Experimental Group (*n*=33)	Control Group (*n*=32)		
**Overall sleep quality**				2.20	.031
Pretest (week 0)	6.85±2.91	7.61±3.14	6.06±2.45		
Mid-test (week 6)		5.91±2.55	7.28±3.52		
Posttest (week 12)		6.03±3.11	6.91±3.33		
Subjective sleep quality					
Pretest (week 0)		1.36±0.74	1.22±0.61		
Mid-test (week 6)		1.03±0.77	1.06±0.67		
Posttest (week 12)		0.91±0.77	1.19±0.69		
Sleep latency					
Pretest (week 0)		1.64±0.93	1.31±0.90		
Mid-test (week 6)		1.24±0.66	1.31±0.90		
Posttest (week 12)		1.45±1.20	1.59±1.27		
Sleep duration					
Pretest (week 0)		1.15±0.83	0.94±0.88		
Mid-test (week 6)		1.00±0.79	0.94±0.98		
Posttest (week 12)		0.97±0.77	1.06±0.91		
Sleep efficiency					
Pretest (week 0)		0.88±0.93	0.78±0.94		
Mid-test (week 6)		0.97±1.05	1.53±2.60		
Posttest (week 12)		0.94±1.12	1.13±1.16		
Sleep disturbances					
Pretest (week 0)		1.21±0.60	0.97±0.40		
Mid-test (week 6)		1.00±0.25	1.00±0.51		
Posttest (week 12)		0.94±0.61	0.94±0.44		
Use of sleeping medication					
Pretest (week 0)		0.24±0.61	0.19±0.74		
Mid-test (week 6)		0.12±0.33	0.13±0.55		
Posttest (week 12)		0.15±0.57	0.22±0.75		
Daytime dysfunction					
Pretest (week 0)		1.12±0.70	0.66±0.60		
Mid-test (week 6)		0.64±0.70	0.97±0.69		
Posttest (week 12)		0.64±0.55	0.72±0.58		

**Table 3:** Summary of Overall Sleep Quality and Subcomponents Over Time in the Experimental and Control Groups

Sleep Quality Component	Mid-Test		Posttest		Interaction
	*B*	*p*	*B*	*p*	Significance
Overall sleep quality	−2.92	< .001	−2.42	.001	Significant
Subjective sleep quality	−0.18	.476	−0.42	.023	Posttest only
Sleep latency	−0.39	.159	−0.46	.082	Not significant
Sleep duration	−0.15	.596	−0.31	.098	Not significant
Sleep efficiency	−0.66	.206	−0.28	.335	Not significant
Sleep disturbances	−0.24	.166	−0.24	.117	Not significant
Sleep medication use	−0.06	.773	−0.12	.148	Not significant
Daytime dysfunction	−0.80	< .001	−0.55	.002	Significant

In terms of subjective sleep quality, no significant interaction was observed between the experimental group and the mid-test (*B*=−0.18, *p*=.476). However, a significant interaction was noted at the posttest (*B*=−0.42, *p*=.023), indicating significant between-group differences in subjective sleep quality at posttest only.

In terms of sleep latency, no significant interactions were found between the experimental group and either the mid-test (*B*=−0.39, *p*=.159) or posttest (*B*=−0.46, *p*=.082). However, the experimental group experienced a notable decrease in sleep latency between the pretest and the mid-test, followed by a slight increase, whereas the control group exhibited a continuous increase. Although these changes were not statistically significant, the performance of the experimental group was superior to the control group, suggesting a potentially positive effect of TRX training on reducing sleep latency.

In terms of sleep duration, no significant interactions were observed between the experimental group and either the mid-test (*B*=−0.15, *p*=.596) or posttest (*B*=−0.31, *p*=.098). A continuous decline in sleep duration was observed between pretest and posttest in the experimental group, while a continuous increase was observed in the control group. This trend suggests a potentially positive effect of TRX training on increasing sleep duration.

In terms of sleep efficiency, no significant interactions were found between the experimental group and either the mid-test (*B*=−0.66, *p*=.206) or posttest (*B*=−0.28, *p*=.335). No notable changes were observed in the experimental group, while the control group exhibited a significant increase from the pretest to the mid-test followed by a decline from the mid-test to the posttest. These findings indicate TRX training does not impact sleep efficiency significantly.

In terms of sleep disturbance, no significant interactions were found between the experimental group and either the mid-test (*B*=−0.24, *p*=.166) or posttest (*B*=−0.24, *p*=.117). However, the continuous decrease in sleep disturbances between pretest and posttest in the experimental group and no significant change over this period in the control group suggest that TRX training may help reduce sleep disturbances.

In terms of sleep medication use, no significant interactions were observed between the experimental group and either the mid-test (*B*=−0.06, *p*=.773) or posttest (*B*=−0.12, *p*=.148). Sleep medication use in the experimental group significantly decreased between pretest and mid-test but slightly increased at posttest, while the opposite trend was observed in the control group. Overall, the experimental group exhibited a greater reduction in sleep medication use over time, suggesting TRX training may play a positive role in decreasing reliance on sleep medication.

In terms of daytime dysfunction, significant interactions were found between the experimental group and both the mid-test (*B*=−0.80, *p*<.001) and posttest (*B*=−0.55, *p*=.002), indicating TRX training effectively reduced daytime dysfunction. The experimental group exhibited a significant decline in daytime dysfunction between pretest and mid-test, with minimal changes thereafter, while the control group showed an initial increase followed by a decline. The greater overall reduction in the experimental group supports the efficacy of TRX training in mitigating daytime dysfunction. In summary, TRX training demonstrated positive effects in improving overall sleep quality, enhancing subjective sleep quality, and alleviating daytime dysfunction.

## Discussion

The respective demographic profiles of the experimental and control groups in this study reflected intergroup homogeneity. The participants were recruited from a regional teaching hospital that was newly established (5 years before the start of this study) and which had a relatively young nursing workforce with lower years of professional experience and a gender ratio that varied from the national nursing staff distribution (Taiwan Union of Nurses Association, 2023). To contextualize the findings, the demographic and sleep quality characteristics of the sample were compared with those reported in prior studies involving Taiwanese nursing staff. Lin et al. (2015) investigated nursing staff from a regional teaching hospital in New Taipei City with an age range of 20–54 (*M*=31.1, *SD*=7.3) years and an average service duration of 12.4 (*SD*=7.3) years. Their participants reported a mean sleep quality score of 6.6 (*SD*=2.0). In contrast, the participants in this study had a similar mean sleep quality score of 6.85 (*SD*=2.91) despite their having a considerably shorter average service duration (*M*=3.31; *SD*=2.87 years). Similarly, Tsai et al. (2019) examined ICU nursing staff from a regional teaching hospital in southern Taiwan with a mean age of 30.6 (*SD*=6.5) years, 8.0 (*SD*=6.0) years of service, and a slightly higher mean sleep quality score of 7.07 (*SD*=3.36). These comparisons highlight the potential influence of certain demographic variables, for example, age, years of service, and work environment (e.g., ICU vs. general ward), on sleep quality. The participants in this study were generally younger and less experienced, and predominantly (55.4%) worked in general wards, which is generally associated with stress and shift rotation patterns different from those in ICU settings. The variation in sleep quality scores observed across these studies may reflect the combined effects of demographic and occupational differences.

The mean overall sleep quality (CPSQI) score of 6.85 (*SD*=2.91) measured in this study is indicative of poor overall sleep quality. Sleep latency (1.64±0.93) earned the highest score, followed by subjective sleep quality (1.36±0.74) and sleep disturbances (1.21±0.60). Consistent with these findings, Tsai et al. (2019) reported a mean overall sleep quality score of 7.07 (*SD*=3.36) among ICU nursing staff, with subjective sleep quality (1.34±0.70) and sleep latency (1.22±0.85) identified as the primary contributors. These results collectively underscore the prevalent poor sleep quality experienced by nursing personnel.

The results of this study demonstrated that a medium- to high-intensity TRX regimen exceeding 12 weeks significantly improved overall sleep quality in the participants as well as the subcomponents of subjective sleep quality and daytime dysfunction. Chang et al. (2020) implemented a 12-week exercise intervention (three weekly sessions lasting 50 min each) involving muscle strength training and stretching on middle-aged participants. The notable improvements reported in subjective sleep quality, sleep duration, sleep disturbances, and daytime dysfunction align with the results of this study. Also, Jiménez-García et al. (2021) used a 12-week TRX-based medium- and high-intensity interval training program (45-min sessions) on older adults, reporting enhanced sleep quality and reduced fatigue levels, which also align with the results of this study. Moreover, Ai et al. (2022) implemented a 12-week, supervised, diverse exercise program (90-min sessions per week) with middle-aged (40–60 y) participants, reporting improved overall sleep quality, sleep disorders, and sleep efficiency. However, as the 12-week medium- to high-intensity TRX exercise programs implemented in this and other studies have not significantly improved the other subcomponents of sleep quality (i.e., sleep latency, sleep duration, sleep efficiency, sleep disturbances, and sleep medication use), further investigation is warranted.

### Limitations

Although the positive effects of TRX training on sleep quality were elucidated in this study, the mechanisms underlying these effects and other potential health benefits were not fully explored. Based on the nature of TRX exercise, positive effects on muscle strength, muscular endurance, and other fitness indicators may be expected as well. Efforts were made to control the exercise habits of the control group by enforcing a uniform intervention protocol (15 min of daily video-guided exercise and avoidance of other regular exercise). However, due to the restrictions imposed by the COVID-19 pandemic, full monitoring and control of all potential confounding factors affecting sleep outcomes (e.g., variations in workload or lifestyle) were not possible, representing a major limitation of this study. Future research should include objective monitoring and longitudinal designs to improve internal validity and explanatory power.

Due to limitations on the scope of this study and the singular focus on sleep quality, related fitness indicators were not measured or presented. Furthermore, relevant physiological measurements such as heart rate variability and cortisol levels, which were not used to directly assess the potential pathways by which exercise may influence sleep. A more comprehensive analysis of influencing factors such as heterogeneity among sample characteristics, training duration, and participants’ baseline health status is recommended for future studies. Incorporating additional physiological indicators and a control group may further enhance the external validity of the results. Addressing these limitations in future research may be expected to provide a more comprehensive understanding of the effects of TRX training on sleep quality and other health outcomes.

### Relevance to Clinical Practice

Due to shift work and heavy workloads (Wang et al., 2025), nursing staff in Taiwan, particularly those with exercise aversion or extreme work fatigue, often exhibit low motivation to engage in exercise. To sustain engagement over a 12-week exercise plan, incorporating lively, engaging, and contemporary TRX exercises or leveraging peer encouragement is considered crucial. For individuals with lower exercise motivation, initiating the program using medium-intensity exercises may help foster sustained interest/commitment (Tartibian et al., 2019). In this study, a 12-week TRX intervention was implemented, comprising 2–3 weekly sessions of 60–90 minutes each. The intervention was shown to have a significant and positive influence on overall sleep quality. The TRX sessions, which were scheduled at 10 a.m. and 6 p.m., offered the option of two training options (i.e., medium and high intensity) and allowed participants to select session times that aligned with their current physical and health conditions.

### Conclusions

Based on the findings of this study, a professionally supervised TRX exercise regimen comprising two 90-min sessions per week has the potential to significantly enhance sleep quality in nurses within 6 weeks of program engagement. Furthermore, continuous training over 12 weeks consistently reinforces these positive effects. The results support the promotion of TRX-based exercise programs as part of hospital efforts to improve sleep quality in nurses to ensure their ability and capacity to provide consistent, high-quality patient care.
